# Recurrence patterns and prognosis of esophageal cancer patients based on the Rbr resection status

**DOI:** 10.3389/fonc.2025.1615285

**Published:** 2025-09-19

**Authors:** Peng Zhang, Guoqing Zhang, Kaishang Zhang, Yujin Qiao, Xiangnan Li, Song Zhao

**Affiliations:** ^1^ Department of Thoracic Surgery, First Affiliated Hospital of Zhengzhou University, Zhengzhou, Henan, China; ^2^ Henan Medical Key Laboratory of Thoracic Oncology, Zhengzhou, Henan, China; ^3^ Henan Province Engineering Research Center of Molecular Pathology and Clinical Experiment of Thoracic Diseases, Zhengzhou, Henan, China

**Keywords:** Rbr, esophageal squamous cell carcinoma, recurrence, locoregional recurrence, neoadjuvant therapy

## Abstract

**Introduction:**

Despite achieving complete resection (R0) and pathological complete response (pCR) in esophageal squamous cell carcinoma (ESCC) patients, recurrence is still observed, resulting in poorer overall survival (OS). We introduced a resection status classification, Rbr^+/-^, which complements the R0 classification.

**Materials and methods:**

We retrospectively reviewed ESCC patients who underwent neoadjuvant therapy plus curative surgery in the Department of Thoracic Surgery at the First Affiliated Hospital of Zhengzhou University between April 2017 and August 2023. Overlap weighting (OW) was used to balance the baseline characteristics between Rbr^+^ and Rbr^-^. Logistic and Cox regression models were conducted to evaluate the association of Rbr status with locoregional recurrence (LRR) and LRR-free survival (LRRFS) and overall survival (OS).

**Results:**

In total, 443 eligible patients were included and categorized into Rbr^+^ (141 patients) and Rbr^-^ (302 patients) groups. After OW, LRR remained significantly higher in the Rbr^+^ group (20.1% *vs* 11.4%, p =0.034, SMD = 0.242). Multivariable logistic regression revealed that Rbr^+^ was associated with the higher risk of LRR (p=0.018, OR: 2.19, 95% CI: 1.14-4.17). The Kaplan-Meier (K-M) curve revealed a worse LRRFS (log-rank p=0.018) in the Rbr^+^ group. According to the multivariable Cox regression analysis, Rbr^+^ was significantly associated with poor LRRFS (p = 0.016, HR: 2.15, 95% CI: 1.15–4.01) but not OS (p = 0.120, HR: 1.52, 95% CI: 0.90-2.58).

**Conclusions:**

Rbr^+^ is associated with a higher LRR rate and poorer LRFS but not OS. A prospective study is necessary to further validate these findings.

## 1 Introduction

Multimodal treatment, including neoadjuvant chemotherapy (NACT) with or without radiotherapy (NACRT) followed by curative surgery, has become the standard of care for locally advanced esophageal squamous cell carcinoma (ESCC). This approach can downstage tumors, increase the likelihood of complete resection, and improve survival outcomes (1–4). Despite the uncertain survival benefits, traditional neoadjuvant therapy combined with programmed death-1 (PD-1) or programmed death-ligand 1 (PD-L1) inhibitors (neoadjuvant chemoimmunotherapy, NACI) has shown significant antitumor effects in recent years. This combination has led to a considerable pathological complete response (pCR) and acceptable toxicity, providing a promising alternative for treating this challenging disease (5–8). However, improved pCR rates have not consistently translated into improved survival benefits, as evidenced by results from several prospective randomized clinical trials (RCTs) (2, 6). It has been reported that recurrence occurs in 10–30% of those who achieve pCR (9–11). In a review of 3154 ESCC patients treated with NACT followed by surgery, Kitagawa et al. highlighted a strong correlation between recurrence-free survival (RFS) and overall survival (OS), underscoring the importance of addressing recurrence and the urgent need to explore alternative strategies to address this challenge.

Achieving complete resection of the esophagus with negative margins (R0 resection) is essential for esophagectomy. The margins encompass the proximal, distal, and circumferential resection margins (CRMs). While a 5-cm longitudinal margin is generally considered safe, the definition and implications of a positive CRM remain uncertain and debated (12, 13). In the context of neoadjuvant therapy, tumor regression often results in the formation of a scar or mass that can adhere tightly to surrounding tissues. In cases with random regression patterns (14–16), scattered tumor cells may remain in the mediastinum, potentially leading to locoregional recurrence (LRR), even when a negative CRM is reported.

Previously, we proposed a novel resection status, Rbr^+/-^, which complements the R0 status of the Royal College of Pathologists (RCP) standards in patients after neoadjuvant therapy (7). In this pilot prospective study, we assessed the efficacy of Rbr^+/-^ in predicting LRR in patients with borderline resectable ESCC (Br-ESCC) (7). Preliminary results revealed that none of the patients (0/7) who underwent Rbr^-^ resection experienced LRR, suggesting that this grading system may serve as a potential predictor for recurrence and could guide postoperative treatment (7). Here, we describe a retrospective study aimed at exploring the relevance of Rbr^+/-^ and LRR and providing a foundation for future research, including our registered prospective clinical trial aimed at exploring the value of adjuvant radiotherapy in patients with Rbr^+^ status (ChiCTR2400082101).

## 2 Materials and methods

### 2.1 Study design and patient selection

This single-center retrospective study was conducted in the Department of Thoracic Surgery at the First Affiliated Hospital of Zhengzhou University, where we reviewed the data of patients who underwent esophagectomy between April 2017 and August 2023. Patients were included in this study if they met the following criteria: (1) had a histologically confirmed diagnosis of ESCC; (2) received any neoadjuvant therapy, regardless of the treatment strategy used; (3) achieved R0 resection; and (4) had available postoperative follow-up data. The exclusion criteria were as follows: (1) a history of previous or concomitant malignancies; (2) lack of neoadjuvant therapy; (3) noncurative resection; (4) pathological confirmation of a cancer type other than ESCC; (5) open surgery; and (6) incomplete medical or follow-up records. The patient characteristics of interest included age, sex, history of alcohol and tobacco use, type of neoadjuvant therapy received, surgery type, clinical stage, postoperative therapy, tumor location, ypStage (pathological stage after neoadjuvant therapy), tumor regression grade (TRG), and Rbr^+/-^. After application of the inclusion and exclusion criteria, eligible patients were categorized on the basis of their Rbr status, and subsequent analyses were conducted accordingly.

This retrospective study was reviewed and approved by the Institutional Review Board and Ethics Committee of the First Affiliated Hospital of Zhengzhou University (approval number: 2024-KY-1392-001). Since this study was retrospective, obtaining written informed consent from patients was not necessary. This study was conducted in accordance with the principles of the Declaration of Helsinki.

### 2.2 Neoadjuvant therapy regimen and surgical procedure

In this study, all patients received a basic regimen of 2–4 cycles of NACT, primarily consisting of platinum-based agents combined with either paclitaxel or docetaxel, administered every three weeks. The typical NACT regimens included cisplatin (75 mg/m²) on day 1, followed by nab-paclitaxel (260 mg/m² on day 1 or 130 mg/m^2^ on days 1 and 8) or docetaxel (75 mg/m²) in combination with cisplatin (75 mg/m²) on day 1 every 21 days. If radiotherapy was administered (NACRT), it consisted of a total dose of 40–45 Gy delivered either simultaneously or sequentially. For NACI, one of the following PD-1 monoclonal antibodies was used every three weeks: camrelizumab (200 mg), pembrolizumab (200 mg), sintilimab (200 mg), tislelizumab (200 mg), or toripalimab (240 mg).

Experienced thoracic surgeons conducted minimally invasive surgery via a minimum of two-field lymphadenectomies 3 to 6 weeks after NACT/NACI or 4 to 8 weeks after NACRT if the tumor was resectable. Three-field lymph nodes dissection (including cervical lymph nodes dissection) was performed when positive supraclavicular lymph nodes were detected pathologically.

### 2.3 Clinical and pathological assessment

Every patient suspected of having esophageal carcinoma underwent esophagoscopy and pathologic examination for diagnosis. Endoscopic ultrasonography (EUS), thoracoabdominal enhanced computed tomography (CT), positron emission tomography (PET) (a small minority) and cervical ultrasonography were utilized to evaluate the clinical stage before surgery. Cervical enhanced CT scans and ultrasound/CT-guided biopsy were conducted if the cervical lymph nodes were suspected to be metastatic. Tumors were staged according to the eighth edition of the American Joint Committee on Cancer/International Union Against Cancer (AJCC/UICC) classification. TRG was assessed by pathologists specializing in gastrointestinal tumors according to the standards set forth by the American Society of Clinical Oncology and the College of American Pathologists (ASCO-CAP) guidelines (**Supplementary Table 1**).

### 2.4 Rbr^+/-^

The content of Rbr^+/-^ has been described in detail in our previous study (7). Representative intraoperative images of Rbr^+^ and Rbr^−^ are presented in **Supplementary Figure 1** and **Supplementary Figure 2**, respectively. It is important to note that the Rbr^+/-^ status, determined intraoperatively after neoadjuvant therapy, was assessed by two experienced thoracic surgeons, with a third senior surgeon resolving any disagreements. All assessments were documented in the surgical records. Rbr^+^ was defined by the absence of a clear boundary between the residual mass/scar and the outer aortic/tracheal membrane, specifically, visual fusion of fibrotic tissue without a distinct separation plane and significant resistance to blunt dissection, combined with no gross tumor on the outer membrane (**Supplementary Figure 1**) and a pathologically negative circumferential margin. Rbr^–^ was defined by a visible fibrous cleavage plane permitting complete blunt mobilization of the membrane from the mass/scar (**Supplementary Figure 2**), no residual tumor on the membrane, and a negative circumferential margin.

### 2.5 Follow-up and endpoints

A significant proportion of patients in this study were enrolled in clinical studies conducted at our center, and adjuvant therapy was initiated either following reassessment by multidisciplinary team (MDT) or in accordance with the specific clinical trial protocols to which they were assigned. The follow-up data were extracted from our prospective database. Patients were scheduled for follow-up visits every 3 to 6 months during the first two years, then every 6 to 12 months from years three to five, and annually thereafter until death or loss to follow-up. The primary endpoint was the incidence of LRR. LRR was defined as the reappearance of a tumor within the anastomotic site and/or in the mediastinum of the surgical field, esophageal bed, and/or regional lymph nodes, including the cervical and para-aortic nodes. The secondary endpoints were OS and LRR-free survival (LRRFS). LRRFS and OS were calculated from the date of surgery until LRR and death or censoring, respectively. In this study, we also reported on distant metastasis (DM), defined as recurrence occurring outside the LRR.

### 2.6 Statistical analysis

Descriptive statistics were used to summarize the baseline characteristics. Continuous variables (age and BMI) were categorized into two groups, and thus, all measures were expressed as frequencies and percentages. A p value of <0.05 was considered to indicate statistical significance. All the statistical tests were two-tailed. Statistical analyses were performed via R software (version 4.4.1, https://www.r-project.org).

All samples were stratified according to Rbr status. To minimize the influence of confounding factors while maximizing the retention of a valid sample size, we initially applied the overlap weighting (OW)-adjusted statistical model to balance the baseline characteristics between the Rbr^+^ and Rbr^-^ groups. Balance was assessed by means of the standardized mean difference (SMD). Variables with an SMD greater than 0.1 indicate insufficient balance and are thus incorporated into the OW. A logistic regression model including unbalanced variables was used to generate propensity scores (PSs), and the weights were calculated as follows: Rbr^-^ = PS, Rbr^+^ = 1-PS. Fisher’s exact test was conducted to compare the LRR and DM between the Rbr^+^ and Rbr^-^ groups. Adjusted regression was performed to further validate the association between Rbr and the incidence of LRR while controlling for confounding factors (age, sex, neoadjuvant therapy, clinical stage, ypStage, TRG, neural invasion, lymphovascular invasion, extent of lymph node dissection and adjuvant therapy). Adjusted Kaplan–Meier (KM) survival curves with the log-rank test and Cox proportional hazards models incorporating covariates used in the PS calculation were used to detect differences in LRRFS and OS. Odds ratios (ORs) and hazard ratios (HRs) with corresponding 95% confidence intervals (CIs) are reported.

## 3 Result

### 3.1 Overall patient cohort and baseline characteristics

**Figure 1:**
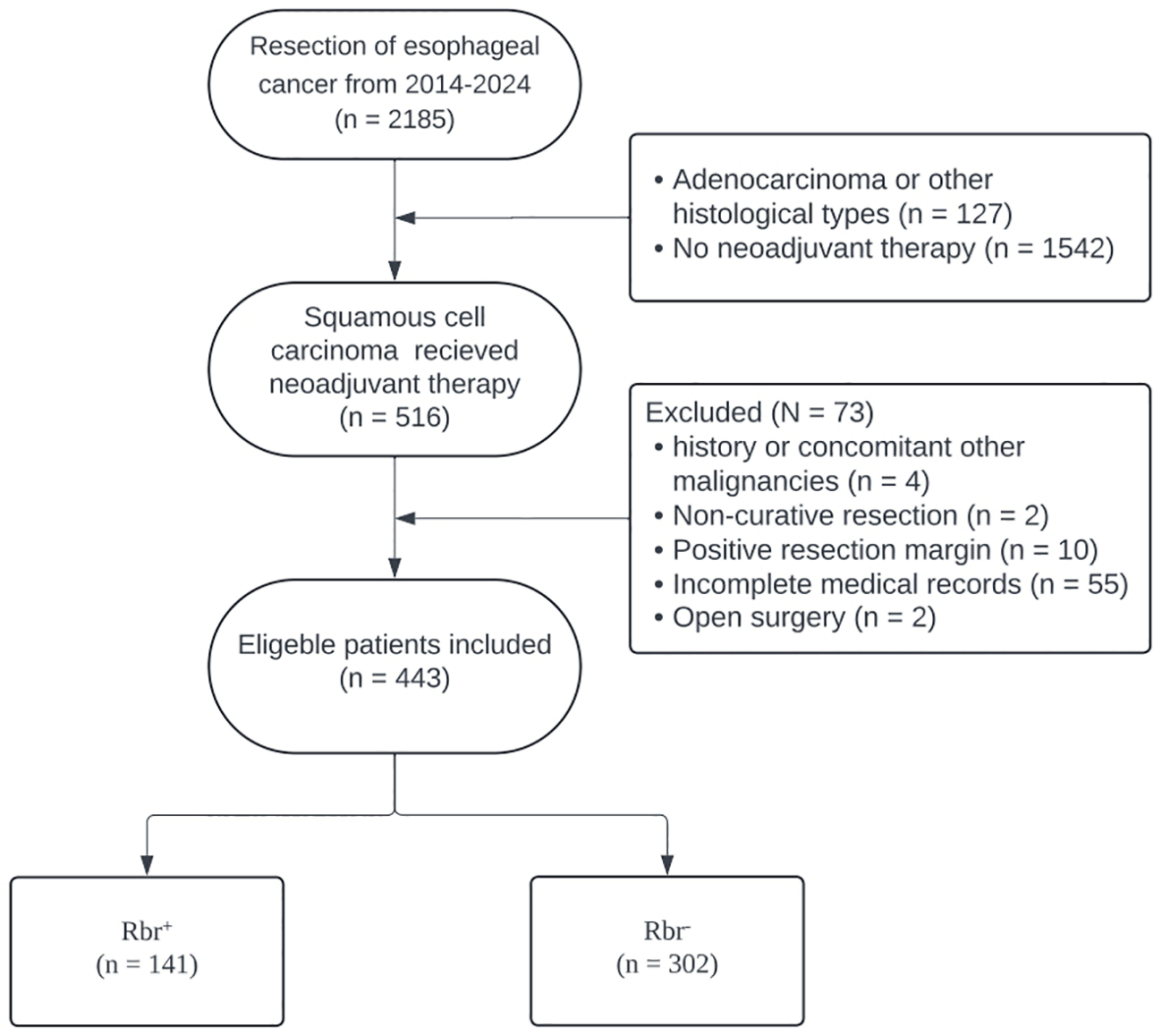
Flow chart of reviewing eligible patients and development of cohorts in this study.

After applying the inclusion and exclusion criteria, a total of 443 eligible patients were included in the study (**Figure 1**). Most patients in this study were over 60 years old (350 individuals, 79%), and the majority were male (318 patients, 71.8%). The mean number of lymph nodes retrieved was 30. Patients were subsequently categorized as Rbr^+^ (141 patients) or Rbr^-^ (302 patients). The baseline characteristics of the patients are presented in **Table 1**. Before OW, only BMI was comparable between the two groups (SMD = 0.014). The proportion of younger patients in the Rbr^+^ group was higher than that in the Rbr^-^ group(age <60 years: 24.1% *vs*. 19.5%), and the percentage of male patients was higher (78.7% *vs*. 68.5%). Advanced disease stages, including clinical stage IVA (23 [16.3%] *vs*. 34 [11.3%]), ypStage II (24 [17.0%] *vs*. 40 [13.2%]), ypStage III (69 [48.9%] *vs*. 97 [32.1%]) and ypStage IV (6 [4.3%] *vs*. 6 [2.0%]), as well as neural invasion (30 [21.3%] *vs*. 36 [11.9%]) and lymphovascular invasion (38 [27%] *vs*. 42 [13.9%]), were more commonly observed in the Rbr^+^ group. In terms of lymph node dissection, three-field node dissection was more prevalent in the Rbr^-^ group than in the Rbr^+^ (71 [23.5%] *vs*. 17 [12.1%]).

**Table 1:** Baseline characteristics of Rbr^+/-^ before and after overlapping weighting.

Variables	Overall (n = 443)	Unweighted			Weighted^a^		
		Rbr^-^ (n = 302)	Rbr^+^ (n = 141)	SMD	Rbr^-^	Rbr^+^	SMD
Age, n (%)							<0.001
≥60	350 (79.0)	243 (80.5)	107 (75.9)	**0.111**	24.8	24.8	
<60	93 (21.0)	59 (19.5)	34 (24.1)		75.2	75.2	
Sex, n (%)				**0.233**			<0.001
Female	125 (28.2)	95 (31.5)	30 (21.3)		23.7	23.7	
Male	318 (71.8)	207 (68.5)	111 (78.7)		76.3	76.3	
BMI (kg/m^2^), n (%)				0.014			0.001
>24.0	199 (44.9)	135 (44.7)	64 (45.4)		46.3	46.2	
≤24.0	244 (55.1)	167 (55.3)	77 (54.6)		53.7	53.8	
Smoke, n (%)				**0.149**			
Never	267 (60.3)	189 (62.6)	78 (55.3)		58.1	58.1	<0.001
Former	46 (10.4)	29 (9.6)	17 (12.1)		9.7	9.7	
Current	130 (29.3)	84 (27.8)	46 (32.6)		32.1	32.1	
Drink, n (%)				**0.139**			<0.001
Never	321 (72.5)	223 (73.8)	98 (69.5)		72.1	72.1	
Former	35 (7.9)	25 (8.3)	10 (7.1)		6.5	6.5	
Current	87 (19.6)	54 (17.9)	33 (23.4)		21.4	21.4	
Neoadjuvant therapy, n (%)				**0.316**			<0.001
NACT	203 (45.8)	133 (44.0)	70 (49.6)		50.6	50.6	
NACI	223 (50.3)	164 (54.3)	59 (41.8)		45.5	45.5	
NACRT	17 (3.8)	5 (1.7)	12 (8.5)		3.9	3.9	
Tumor location, n (%)				**0.213**			<0.001
Upper	33 (7.4)	21 (7.0)	12 (8.5)		7.9	7.9	
Middle	271 (61.2)	177 (58.6)	94 (66.7)		64.5	64.5	
Lower	139 (31.4)	104 (34.4)	35 (24.8)		27.6	27.6	
Clinical stage, n (%)				**0.164**			<0.001
II	236 (53.3)	167 (55.3)	69 (48.9)		51.5	51.5	
III	150 (33.9)	101 (33.4)	49 (34.8)		34.9	34.9	
IVA	57 (12.9)	34 (11.3)	23 (16.3)		13.6	13.6	
Surgery, n (%)				**0.203**			<0.001
McKeown	426 (96.2)	287 (95.0)	139 (98.6)		98.0	98.0	
Others^b^	17 (3.8)	15 (5.0)	2 (1.4)		2.0	2.0	
ypStage, n (%)				**0.485**			<0.001
I	201 (45.4)	159 (52.6)	42 (29.8)		37.4	37.4	
II	64 (14.4)	40 (13.2)	24 (17.0)		16.4	16.4	
III	166 (37.5)	97 (32.1)	69 (48.9)		43.2	43.2	
IV	12 (2.7)	6 (2.0)	6 (4.3)		3.0	3.0	
Differentiation, n (%)				**0.172**			<0.001
Well	94 (21.2)	70 (23.2)	24 (17.0)		17.8	17.8	
Moderate	288 (65.0)	194 (64.2)	94 (66.7)		67.6	67.6	
Poor	61 (13.8)	38 (12.6)	23 (16.3)		14.7	14.7	
TRG, n (%)				**0.475**			<0.001
0	118 (26.6)	94 (31.1)	24 (17.0)		20.2	20.2	
1	72 (16.3)	56 (18.5)	16 (11.3)		13.8	13.8	
2	125 (28.2)	81 (26.8)	44 (31.2)		31.2	31.2	
3	128 (28.9)	71 (23.5)	57 (40.4)		34.8	34.8	
Neural invasion, n (%)				**0.253**			<0.001
Yes	66 (14.9)	36 (11.9)	30 (21.3)		18.2	18.2	
No	377 (85.1)	266 (88.1)	111 (78.7)		77.8	77.8	
Lymphovascular invasion, n (%)				**0.328**			<0.001
Yes	80 (18.1)	42 (13.9)	38 (27.0)		22.2	22.2	
No	363 (81.9)	260 (86.1)	103 (73.0)		77.8	77.8	
Extent of lymph node dissection, n (%)				**0.303**			<0.001
Two-field	355 (80.1)	231 (76.5)	124 (87.9)		84.6	84.6	
Three-field	88 (19.9)	71 (23.5)	17 (12.1)		15.4	15.4	
Adjuvant chemotherapy, n (%)				**0.205**			<0.001
Radiotherapy included^c^	27 (6.1)	15 (5.0)	12 (8.5)		7.4	7.4	
Others^d^	243 (54.9)	161 (53.3)	82 (58.2)		56.6	56.6	
None	173 (39.1)	126 (41.7)	47 (33.3)		36.0	36.0	

BMI, body mass index; NACT, neoadjuvant chemotherapy; NACRT, neoadjuvant chemoradiotherapy; NACI, neoadjuvant chemoimmunotherapy; TRG, tumor regression grade. SMD>0.1, denoting imbalance between groups, are highlighted in bold.

a After overlap weighting, a single individual no longer represents a single data entity; thus, raw counts are not reported after overlap weighting.

b In addition to McKeown surgery, we also performed Ivor–Lewis esophagectomy and inflatable video-assisted mediastinoscopy-assisted transhiatal esophagectomy.

c Including chemoradiotherapy and immunoradiotherapy.

d Without radiotherapy, adjuvant therapy typically consists of chemotherapy alone or a combination of chemotherapy and immunotherapy.

After OW, for unbalanced variables (age, sex, smoking status, alcohol consumption, neoadjuvant therapy, tumor location, clinical stage, surgery type, ypStage, differentiation, TRG, neural invasion, lymphovascular invasion, extent of lymph node dissection and adjuvant therapy), the distributions of all baseline covariates were well balanced and comparable. Subsequent analyses were conducted on the basis of these balanced data.

### 3.2 Outcome of LRR and patterns of LRR and DM

**Figure 2:**
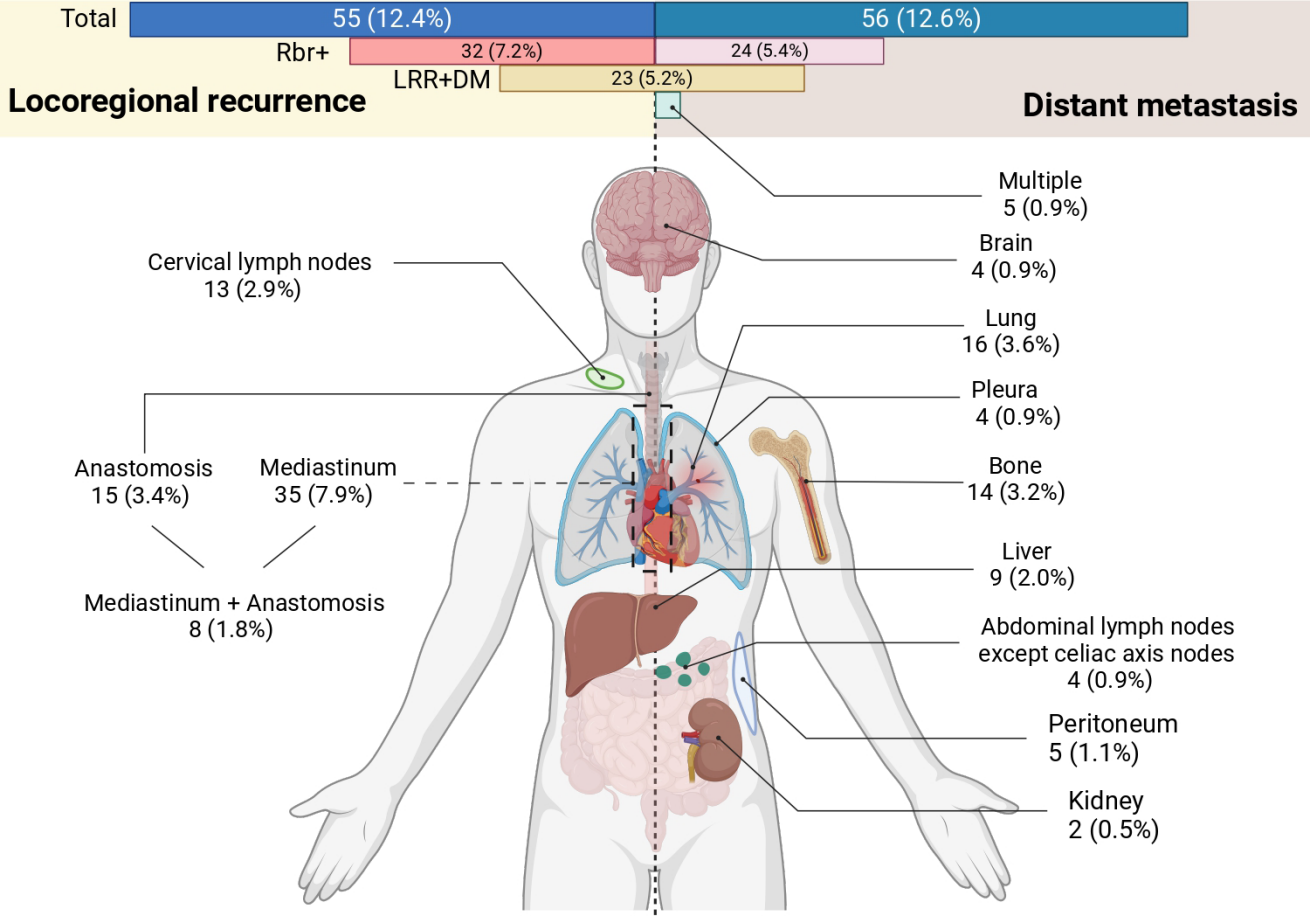
Patterns of locoregional and distant recurrence observed in this study. All percentages in this figure have been recalculated based on the entire cohort. Created in BioRender. zhang, p. (2024) BioRender.com/b93k052. LRR, locoregional recurrence; DM, distant metastasis.

Before OW, the incidences of LRR and DM were similar, at 12.4% and 12.6%, respectively, in the total cohort (**Table 2**). However, LRR was the primary pattern of recurrence in the Rbr^+^ group compared with DM (32 [22.7%] *vs*. 24 [17.0%] (**Table 2**; The incidence of the total cohort is shown in **Figure 2**). LRR was more frequently observed in the Rbr+ group (32 [22.7%] vs 23 [7.6%], p<0.001, SMD=0.430). Following OW, the incidence of LRR remained significantly higher in the Rbr^+^ group (20.1% *vs*. 11.4%, p = 0.034, SMD = 0.24) (**Table 2**).

**Table 2:** Comparison of locoregional recurrence rates between the Rbr^-^ and Rbr^+^ groups before and after weight overlap.

Variables	Overall	Unweighted				Weighted^a^			
		Rbr^-^ (n = 302)	Rbr^+^ (n = 141)	P	SMD	Rbr^-^	Rbr^+^	P	SMD
Locoregional recurrence				**<0.001**	**0.430**			**0.034**	**0.242**
Yes	55 (12.4)	23 (7.6)	32 (22.7)			11.4	20.1		
No	388 (87.6)	279 (92.4)	109 (77.3)			88.7	79.9		
Distant metastasis									
Yes	56 (12.6)	32 (10.6)	24 (17.0)	0.081	**0.187**	14.7	15.9	0.774	0.032
No	387 (87.4)	270 (89.4)	117 (83.0)			85.3	84.1		

SMD>0.1 or P<0.05, denoting imbalance between groups, are highlighted in bold.

a After overlap weighting, a single individual no longer represents a single data entity; thus, raw counts are not reported after overlap weighting. SMD, standardized mean difference.

Multivariate logistic regression analysis revealed that only Rbr^+^ was significantly associated with LRR (p = 0.018, OR: 2.19, 95% CI: 1.14-4.17) (**Table 3**). Additionally, lymphovascular invasion tended to increase the incidence of LRR (p = 0.099, OR: 2.15, 95% CI: 0.87–5.34).

**Table 3:** Multivariable logistic regression analysis of factors relevant to LR.

Variables	OR	95% CI	P value
Age			
<60	1.00		
≥60≥	0.89	0.42–1.88	0.749
Sex			
Female	1.00		
Male	1.63	0.67–3.97	0.279
Rbr			
Rbr-	1.00		
Rbr+	2.19	1.14–4.17	**0.018**
Clinical stage			
II	1.00		
III	1.82	0.83–3.99	0.132
IVA	1.82	0.68–4.85	0.232
Neoadjuvant therapy			
NACT	1.00		
NACI	0.60	0.29–1.23	0.164
NACRT	2.13	0.52–8.62	0.290
TRG			
0	1.00		
1	1.46	0.44–4.84	0.535
2	0.83	0.21–3.21	0.784
3	1.15	0.31–4.27	0.839
ypStage			
I	1.00		
II	0.55	0.15–2.01	0.367
III	1.17	0.44–3.12	0.760
IV	2.97	0.67–13.18	0.151
Extent of lymph node dissection			
Two-field	1.00		
Three-field	0.43	0.12–1.57	0.201
Neural invasion			
No	1.00		
Yes	1.94	0.81–4.64	0.136
Lymphovascular invasion			
No	1.00		
Yes	2.15	0.87–5.34	0.099
Adjuvant chemotherapy			
None	1.00		
Others	1.65	0.77–3.52	0.196
Radiotherapy included	0.72	0.17–3.07	0.661

NACT, neoadjuvant chemotherapy; NACRT, neoadjuvant chemoradiotherapy; NACI, neoadjuvant chemoimmunotherapy; TRG, tumor regression grade; OR, odds ratio; CI, confidence interval. P<0.05, denoting imbalance between groups, are highlighted in bold.

We also reported the patterns of recurrence in this study (**Figure 2**). The recurrence rates for LRR and DM were similar, with LRR in 55 patients (12.4%) and DM in 56 patients (12.6%). Additionally, 23 patients (5.25%) experienced both LRR and DM simultaneously. With regard to LRR, the mediastinum was the predominant site of recurrence (35 patients, 7.9%), followed by the anastomosis (15 patients, 3.4%) and cervical lymph nodes (13 patients, 2.9%). In terms of DM, the lung (16 patients, 3.6%) and bone (14 patients, 3.2%) were the most common sites. Furthermore, 5 individuals (0.9%) had multiple sites of DM.

### 3.3 The outcomes of LRRFS and OS for Rbr^+^ patients

**Figure 3:**
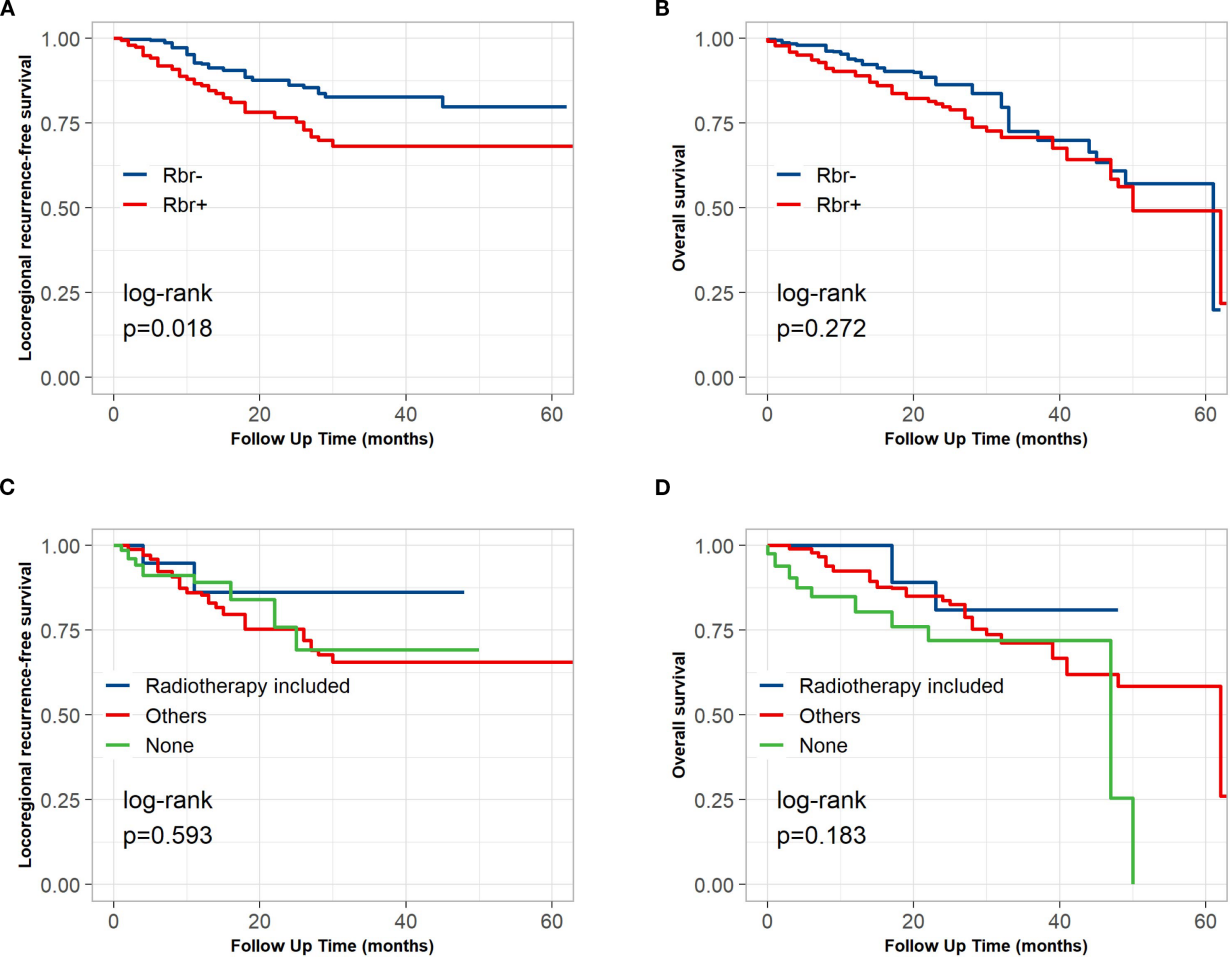
Locoregional recurrence-free (LRRFS) and overall survival (OS) of Rbr+/- status in the whole cohort and adjuvant therapy (AT) in Rbr+ group. **(A)** LRRFS stratified by Rbr+ and Rbr-. **(B)** OS stratified by Rbr+ and Rbr-. **(C)** LRRFS stratified by AT. **(D)** OS stratified by AT.

The median follow-up for the entire population from the date of definitive surgery was 18 months (IQR: 9–63 months). The median time to LRR was 16 months (IQR: 8–63 months). The KM curves indicated that Rbr^+^ status was associated with shorter LRFS (p = 0.018, **Figure 3A**) but was not significantly associated with OS (p = 0.272) (**Figure 3B**). Similarly, multivariate Cox regression models were developed and revealed that Rbr^+^ was an independent factor associated with unfavorable LRRFS (p=0.008; HR: 2.56; 95% CI: 1.28–5.11) but that OS was not (p=0.120; HR: 1.52; 95% CI: 0.90–2.58) (**Table 4**). In addition, ypStage IV disease was significantly associated with worse LRRFS (p=0.008, hazard ratio (HR): 5.03, 95% CI: 1.54–16.47) and OS (p=0.004, HR: 5.11, 95% CI: 1.70–15.40). Neural invasion was an adverse factor for LRRFS (p=0.031, HR: 2.25, 95% CI: 1.08–4.71) (**Table 4**).

**Table 4:** Multivariable Cox regression analysis of factors relevant to LR.

Variables	LRRFS			OS		
	HR	95% CI	P value	HR	95% CI	P value
Age						
<60	1.00			1.00		
≥60	0.69	0.35–1.36	0.28	1.24	0.70–2.20	0.456
Sex						
Female	1.00			1.00		
Male	1.17	0.51–2.72	0.71	1.16	0.62–2.19	0.640
Rbr						
Rbr^-^	1.00			1.00		
Rbr^+^	2.56	1.28–5.11	**0.008**	1.52	0.90–2.58	0.120
Clinical stage						
II	1.00			1.00		
III	1.42	0.72–2.79	0.314	1.13	0.62–2.04	0.693
IVA	1.60	0.62–3.95	0.306	1.36	0.69–2.69	0.375
Neoadjuvant therapy						
NACT	1.00			1.00		
NACI	0.86	0.41–1.84	0.707	0.81	0.41–1.59	0.537
NACRT	2.34	0.58–9.49	0.234	2.26	0.56–9.10	0.251
TRG						
0	1.00			1.00		
1	1.07	0.33–3.46	0.597	1.43	0.50–4.10	0.505
2	0.73	0.19–2.85	0.638	2.02	0.67–6.11	0.214
3	1.13	0.31–4.08	0.851	1.32	0.40–4.33	0.652
ypStage						
I	1.00			1.00		
II	0.72	0.22–2.40	0.594	1.26	0.54–2.95	0.600
III	1.32	0.58–2.98	0.506	1.33	0.67–2.64	0.410
IV	5.03	1.54–16.47	**0.008**	5.11	1.70–15.40	**0.004**
Extent of lymph node dissection						
Two-field	1.00			1.00		
Three-field	0.50	0.13–1.89	0.307	0.98	0.39–2.46	0.972
Neural invasion						
No	1.00			1.00		
Yes	2.25	1.08–4.71	**0.031**	1.20	0.67–2.17	0.540
Lymphovascular invasion						
No	1.00			1.00		
Yes	1.85	0.89–3.85	0.099	1.40	0.79–2.49	0.252
Adjuvant chemotherapy						
None	1.00			1.00		
Others	0.99	0.47–2.12	0.987	0.74	0.28–0.93	**0.028**
Radiotherapy included	0.40	0.09–1.83	0.239	0.51	0.32–1.69	0.471

NACT, neoadjuvant chemotherapy; NACI, neoadjuvant chemoimmunotherapy; NACRT, neoadjuvant radiotherapy; LRRFS, locoregional recurrence-free survival; OS, overall survival; HR, hazard ratio; CI, confidence interval. P<0.05, denoting imbalance between groups, are highlighted in bold.

### 3.4 Subgroup analysis of the role of adjuvant therapy in the Rbr^+^ group

Owing to the significant association between Rbr^+^ and LRR, AT was categorized into three groups: those involving radiotherapy, those involving other treatments (chemotherapy or chemoimmunotherapy), and those with no treatment. This categorization aimed to investigate the role of adjuvant radiotherapy for Rbr^+^ patients in this study. As shown in **Figures 3C, D**, OS (log-rank p=0.183) and LRRFS (log-rank p=0.593) were not significantly associated with AT type. Nevertheless, the median OS of the radiotherapy group was not reached, while the median OS times in the chemotherapy group and no AT group were 62 months (IQR: 30-Inf) and 47 months (IQR: 22–50 months), respectively, suggesting that radiotherapy is a promising option for improving the OS of patients with Rbr^+^.

## 4 Discussion

This observational study presents a resection status, Rbr^+/-^, in ESCC patients following neoadjuvant therapy, building on previously mentioned concepts (7). It distinguishes between Rbr^+^ and Rbr^-^ categories as a supplement to the R0 classifications defined by the RCP standard. Although similar results were preliminarily reported in our previous study (7), the current findings demonstrate for the first time that patients categorized as Rbr^+^ exhibit a significantly higher LRR rate (32 [22.7%] *vs*. 23 [7.6%], p<0.001, SMD = 0.430). OW is a PS method designed to mimic key characteristics of RCTs (17). Even after adjusting for imbalanced variables via OW, the LRR remained significantly higher in the Rbr^+^ group (20.1% *vs*. 11.4%, p=0.034, SMD = 0.242). The multivariable logistic regression model indicated that Rbr^+^ was an independent risk factor for LRR (p=0.018, OR: 2.19, 95% CI: 1.14-4.17). Additionally, the multivariate Cox analysis revealed that Rbr^+^ was associated with poorer LRRFS (p=0.008, HR: 2.56, 95% CI: 1.28-5.11) than Rbr^-^. These findings suggest that Rbr^+^ might serve as a potential predictor of LRR following neoadjuvant therapy and surgery; thus, more intensive monitoring and appropriate adjuvant therapy are promising for patients with Rbr^+^ to improve their survival outcomes.

The mechanism by which Rbr^+^ status is associated with increased LRR despite complete resection (R0) remains unclear. We speculate that random tumor regression may be a contributing factor. Recently, the pattern of tumor regression was assessed in light of a proposed strategy for esophageal preservation following neoadjuvant therapy (14–16), although the findings remain to be interpreted and discussed. In 2013, Lanschot et al. reviewed 71 (70%) esophageal cancer patients whose residual tumor cells remained after NACRT (15). Despite the small proportion, the surrounding stroma with residual tumors accounted for 42% of cases, representing 30 patients (15). Even considering the regressive changes and/or residual tumor invasion into the surrounding stroma (ycT3), which pertains to patients initially involving all layers of the esophageal wall, 21% of the 51 overall patients exhibited a random regression pattern (15). Chuang and colleagues evaluated 76 patients with near pCR and found no rule of tumor regression (14). In addition, their results were inconsistent with those reported by Shapiro and colleagues (14). In contrast to these two studies (14, 15), Wang et al. provided a detailed description of regression patterns specifically in ESCC patients after NACRT. Four types of regression patterns have been proposed, with Type 4 (random regression) showing comparability to Types 1–3 (nonrandom regression) (16), which means that predicting tumor regression patterns remains challenging. On the basis of these results, when Rbr^+^ occurs, residual masses or proliferative tissue may persist in the mediastinum, as we previously demonstrated (7), whereas the resection margin is located in the tumor regression zone, resulting in pathological R0 and potentially contributing to development recurrence.

Generally, DM is reported as the predominant recurrence type of esophageal cancer following neoadjuvant therapy (18–20). However, in this study, LRR remained the primary type of recurrence compared with DM in the Rbr^+^ group (**Table 2**). The significantly advanced ypT stage partially contributed to this outcome. Similar results have been reported in previous studies (7, 21). BRES-1 was our phase 2 study in which the safety and efficacy of NACI were assessed for patients with borderline resectable ESCC (7). Among the patients who underwent R0 resection (18 patients, 81.8%), four experienced LRR, whereas none had DM. When the patients were categorized as Rbr^+^ or Rbr^-^, all recurrences were in the Rbr^+^ group (7). COSMOS is another multicenter phase 2 trial in which NACT was evaluated for initially unresectable locally advanced ESCC (21). After 3 years of follow-up, 7 (37%) of the 19 R0 patients had a recurrence or disease progression, while LRRs (4, 21%) slightly exceeded DMs (3,16%). Notably, in that study, the recurrence patterns in the non-R0 group were also reported, and a total of 29 patients (59%) were noted to have experienced tumor progression. In this group, the rate of LRR was higher than that of DM was (38% *vs*. 21%), further supporting the association between T stage and LRR (21).

In our study, Rbr status did not influence OS. When various AT regimens were compared with regard to OS in the Rbr^+^ group, the KM curves revealed that the median OS for the group receiving radiotherapy had not yet been reached. These findings suggest that appropriate AT might be a promising option for improving OS. Evidence is available from the JCOG1109 NExT clinical trial (2). In this study, LRR was more common in the NeoCF group (38 [38%] of 101) and the NeoCF+D group (33 [43%] of 76) than in the NeoCF+RT group (17 [23%] of 75 patients). Accordingly, radiotherapy or chemoradiotherapy was administered more frequently in the NeoCF and NeoCF+D groups, potentially contributing to improved OS (2).

Given the relatively high recurrence rate after neoadjuvant therapy plus surgery, AT is theoretically necessary. However, optimal patient selection, timing and regimen remain unclear, and previous studies have been inconclusive (22–25). In our cohort, Rbr status itself did not translate into inferior OS (**Table 4**, **Figure 3B**), suggesting that appropriately tailored AT may mitigate the adverse impact of residual disease. Because LRR is more common among Rbr^+^ patients, adjuvant radiotherapy, delivered specifically to the mediastinum and tumor bed, could confer an OS benefit (26, 27). Indeed, we observed a trend toward improved OS in Rbr^+^ patients who received postoperative radiotherapy, although statistical significance was not reached (**Figure 3D**), likely due to the small sample size. Intriguingly, the “other” adjuvant category (chemotherapy alone or combined chemotherapy and immunotherapy) was independently associated with better OS in multivariable Cox analysis, further supporting the notion that adequate AT is critical in this subgroup. However, the trade−off between treatment−related toxicity and long−term benefit demands careful consideration, and the optimal AT regimen has yet to be established. Specifically, the integration of immunotherapy holds considerable promise for reshaping the therapeutic paradigm in ESCC in recent years (28–30). Prospective, large−scale trials are therefore essential to confirm the survival advantages of adjuvant radiotherapy, chemoradiotherapy, and chemo−immunotherapy in Rbr^+^ ESCC.

As a supplementary indicator to standard R0 resection, Rbr^+^ status was associated with a higher incidence of LRR, suggesting that such patients may represent a high-risk subgroup. While current consensus clearly recommends adjuvant therapy for patients with R1 or R2 resection, our findings imply that Rbr^+^ patients, although pathologically R0, might also benefit from closer surveillance and, in selected cases, consideration of adjuvant radiotherapy or intensified systemic therapy. This potential framework positions Rbr as a clinical tool to refine risk stratification and guide postoperative management, pending validation in prospective multicenter studies. Meanwhile, it must be emphasized that histopathological examination remains the gold standard for margin assessment. Rbr is not intended to replace pathology but rather to identify cases warranting heightened pathological scrutiny. We acknowledge that conventional block-based pathology may miss microscopic disease due to sampling limitations, which likely explains instances where intraoperative fibrosis (Rbr^+^) predicts recurrence despite negative margins. Future efforts should therefore also focus on refining pathological protocols, for example through systematic whole-mount sectioning, serial deeper sections, or adjunctive immunohistochemistry, to better capture subclinical tumor extension. Ultimately, bridging this gap with advanced pathology techniques will be essential for more definitive and reproducible margin evaluation.

To maximize the utility of Rbr as a supplementary tool and mitigate its inherent reliance on operator experience and subjective interpretation, future efforts must also focus on standardizing the Rbr assessment itself. Going forward, we plan to create a weighted Rbr index based on quantifiable imaging and intraoperative metrics. For example, we will measure the two−dimensional area of residual mass or fibrosis and assign incremental points for increasing size. Likewise, intraoperative dissection difficulty will be graded on a three−level scale (easy, moderate, difficult) with corresponding score values. In a prospective, multicenter setting, these component weights and threshold cut−points will be optimized and potentially adjusted using the four established pathologic regression patterns as modifiers. Such a structured algorithm will both standardize Rbr assessment across centers and allow automated or semi−automated implementation in future trials.

This study also has several limitations. Firstly, while we adjusted for several important confounders, this study lacked data on other potential variables since the characteristics of retrospective study. Secondly, information bias may be present; for example, patients who experienced recurrence or death during follow-up were more likely to be lost to follow-up or to provide inaccurate endpoint information. Thirdly, it remains uncertain whether the Rbr^+/-^ system is applicable to patients undergoing NACRT, as most participants in this study received NACT. Fourthly, the number of patients who received radiotherapy was relatively small, which warrants confirmation in larger-scale studies. Finally, as a single-center retrospective study, our findings require validation through prospective multicenter trials before clinical implementation.

## Data Availability

The original contributions presented in the study are included in the article/**Supplementary Material**. Further inquiries can be directed to the corresponding authors.
