# Supplementary material for: Recurrence patterns and prognosis of esophageal cancer patients based on the Rbr resection status
Source: Front Oncol. 2025 Sep 19;15:1615285. doi: 10.3389/fonc.2025.1615285 (PMC12491039; doi:10.3389/fonc.2025.1615285)

**Supplemental table 1 Definition of TRG according to the ASCO-CAP guidelines**

| TRG 0 | no residual cancer cells |
| --- | --- |
| TRG 1 | single cancer cell or cancer cell cluster |
| TRG 2 | fibrotic response over residual cancer cells |
| TRG 3 | almost no fibrosis, visible large residual cancer |

TRG, tumor regression grade; ASCO-CAP, the American Society of Clinical Oncology and the College of American Pathologists

**Supplemental figure 1. Representative case of Rbr^+^**. Intraoperative detection revealed fibrous tissue (yellow arrows) between the esophagus (green arrow) and the descending aorta (red arrow), with no clear boundary observed.


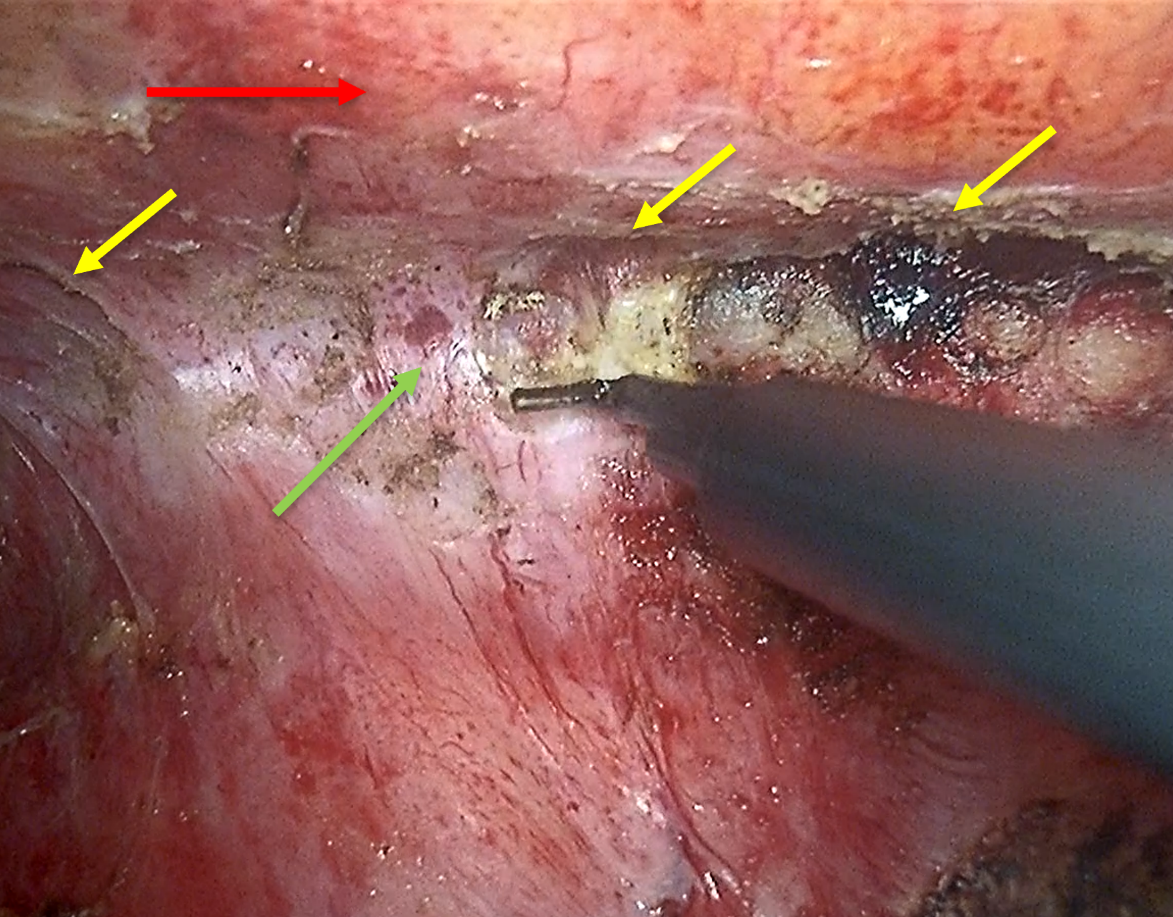


**Supplemental figure 2. Representative case of Rbr^-^**. Intraoperative detection revealed a clear boundary between the esophagus (green arrow) and the descending aorta (red arrow).


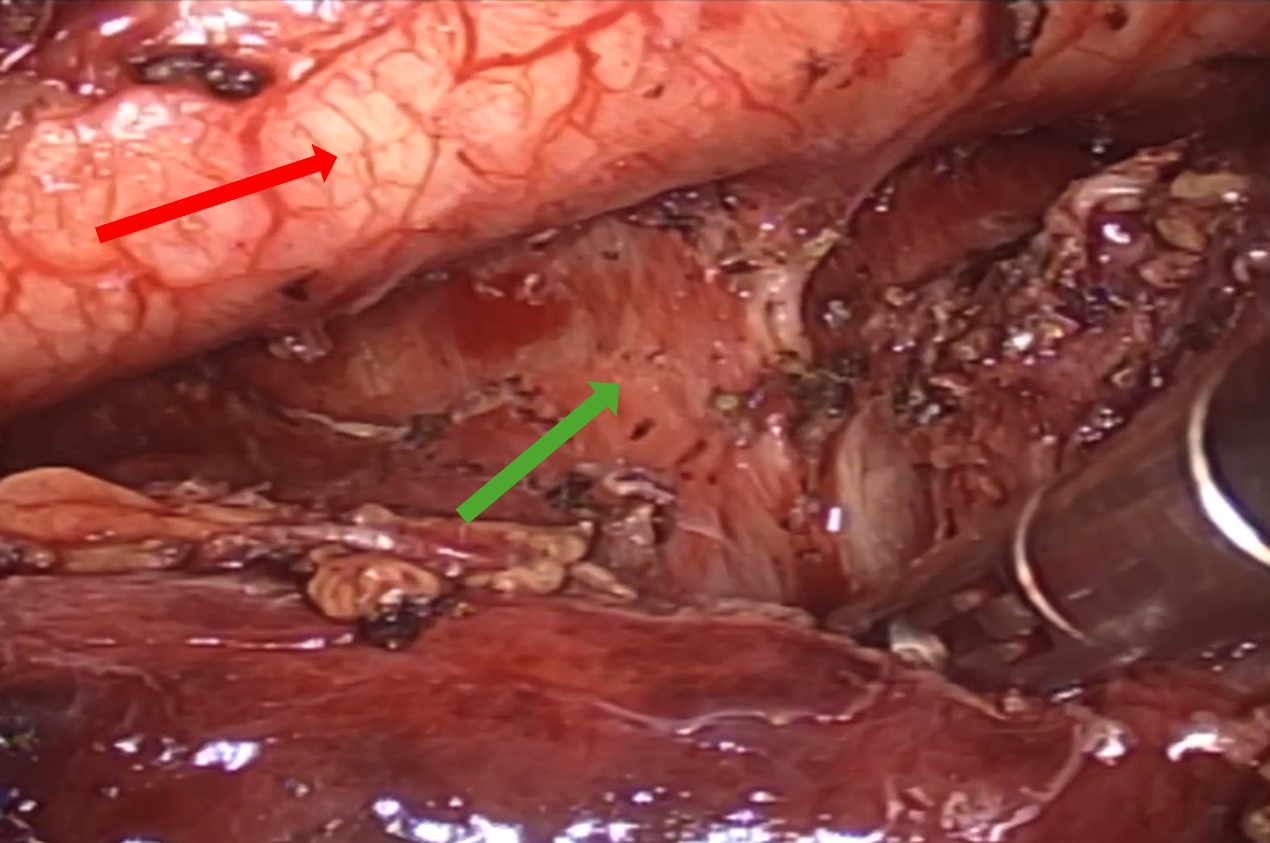

Supplement: Supplementary file 1 [file DataSheet1.docx]
